# Integrative Physiological and Transcriptomic Analysis Reveals the Transition Mechanism of Sugar Phloem Unloading Route in *Camellia oleifera* Fruit

**DOI:** 10.3390/ijms23094590

**Published:** 2022-04-21

**Authors:** Jing Zhou, Bingshuai Du, Yuqing Chen, Yibo Cao, Mingxin Yu, Lingyun Zhang

**Affiliations:** Research & Development Center of Blueberry, Key Laboratory of Forest Silviculture and Conservation of the Ministry of Education, Beijing Forestry University, Beijing 100083, China; zhoujin.1234@163.com (J.Z.); dubingshuai624@163.com (B.D.); chenyuqing@bjfu.edu.cn (Y.C.); caoyibo@bjfu.edu.cn (Y.C.); ymxbjfu@163.com (M.Y.)

**Keywords:** phloem unloading transition, transcriptome analysis, sink organs, callose, plant hormone

## Abstract

Sucrose phloem unloading plays a vital role in photoassimilate distribution and storage in sink organs such as fruits and seeds. In most plants, the phloem unloading route was reported to shift between an apoplasmic and a symplasmic pattern with fruit development. However, the molecular transition mechanisms of the phloem unloading pathway still remain largely unknown. In this study, we applied RNA sequencing to profile the specific gene expression patterns for sucrose unloading in *C. oleifera* fruits in the apo- and symplasmic pathways that were discerned by CF fluoresce labelling. Several key structural genes were identified that participate in phloem unloading, such as *PDBG11*, *PDBG14*, *SUT8*, *CWIN4,* and *CALS10*. In particular, the key genes controlling the process were involved in callose metabolism, which was confirmed by callose staining. Based on the co-expression network analysis with key structural genes, a number of transcription factors belonging to the MYB, C2C2, NAC, WRKY, and AP2/ERF families were identified to be candidate regulators for the operation and transition of phloem unloading. KEGG enrichment analysis showed that some important metabolism pathways such as plant hormone metabolism, starch, and sucrose metabolism altered with the change of the sugar unloading pattern. Our study provides innovative insights into the different mechanisms responsible for apo- and symplasmic phloem unloading in oil tea fruit and represents an important step towards the omics delineation of sucrose phloem unloading transition in crops.

## 1. Introduction

In plants, the products of photosynthesis are delivered from photoautotrophic tissues to distant sink organs, such as fruits and roots, where they are utilized for growth or storage [1,2]. Sucrose (Suc) is the principal transport form of photoassimilates in most plants. The aims of modern agriculture are to maximize the amount of carbon-based photoassimilates allocated to sink organs by the phloem. It is now generally believed that phloem unloading plays a key role in partitioning photoassimilates, hence, determining plant productivity and fruit quality to a large extent [2]. Therefore, it is critical to understand the sucrose phloem unloading patterns to improve fruit quality and yield.

*Camellia oleifera* (oil tea) is a kind of crop used to extract high quality edible oil, which originated from China. The oil from its fruit is beneficial to human health containing over 80% monounsaturated fatty acids [3,4,5]. However, the low yield restricted the development of the tea oil industry. It is important to understand the phloem unloading of photoassimilates in the fruit of oil tea to improve the yield. We previously reported the switch of the phloem unloading pathway between an apoplasmic and symplasmic pattern during *C. oleifera* fruit development [6]. However, the molecular mechanism and transcriptional regulation about the transition of the phloem unloading pathway in oil tea is still unclear.

Phloem unloading contains two processes: transport across the boundary of the sieve element–companion cell (SE-CC) complex, and subsequently the transfer to the sink parenchyma cells. There are two kinds of different patterns for sucrose phloem unloading: the symplasmic pathway through interconnecting plasmodesmata (PD), and the apoplasmic pathway across the surrounding cell wall matrix. Sucrose can be unloaded by symplasmic transport, the apoplasmic pathway, or a combination of both. In different plant species, the routes of phloem unloading varies. Even in the same plant, the Suc unloading pathway differs according to not only types but also the developmental stages of sinks [7], and the corresponding transport mechanisms and characteristics are also altered. For example, in tomatoes (*Solanum lycopersicon*), the phloem transport changes from the symplasmic to the apoplasmic pathway when the content of hexose begins to accumulate largely in the fruit pericarp [8]. In grapes, when the fruit ripens, accompanied with the accumulation of hexose (*Vitis vinifera*), the symplasmic unloading route is converted to the apoplasmic pathway [9]. In addition, in the fruit’s peaches (*Prunus persica*) and jujubes (*Ziziphus jujuba*), the unloading pathway even changed twice, from the apoplasmic route at the early developmental stage to the symplasmic pathway at the middle phase and back to an apoplasmic pattern late in development [10,11,12,13,14]. In addition to fleshy fruit, in fruit with the seed as the main harvesting organ, the apoplasmic unloading pathway is usually adopted in seeds due to the symplasmic isolation between filial and maternal tissues; however, the unloading pattern in the pericarp is variable. For example, in *Xanthoceras sorbifolia*, the symplasmic phloem-unloading pathway is interrupted by the apoplasmic route in the pericarp with the development of fruit [15]. Although the shift of the phloem unloading pathway has been reported in an increasing number of species, the physiological and molecular mechanism behind it and how to trigger the switch still remains largely unknown.

In the symplasmic pathway, Suc unloading requires functional plasmodesmata (PD) connecting the phloem and the surrounding parenchyma cells. Callose deposited at the PD neck region plays a regulatory role in modulating the permeability of the PD channel [16]. Callose is a kind of polysaccharide synthesized in the cell wall and deposited at the neck region of the PD, which squeezes the cytoplasmic channel inwardly, and results in a reduction of free space for molecular substances to pass through the PD channel [17,18,19]. The previous studies showed that PD-related proteins, such as Callose synthase (CALS), β-1,3-glucanase (PDBG), and PD-associated callose binding protein (PDCB), alter the PD permeability and even the phloem unloading pathway [20] via maintaining callose homeostasis at the PD zone [21,22,23,24,25]. 

In the apoplasmic pathway, the exit of Suc from the SE–CC complex into the apoplasmic space is possibly facilitated by SWEETs, a newly discovered family of Suc uniporters [26]. Suc in the apoplasmic space can be either assimilated directly into phloem parenchyma cells (PCs) via sucrose transporters (SUTs), or firstly catalyzed into hexose by cell wall invertases (CWINs), and then transferred into the recipient sink PCs by hexose transporters (HTs) [27,28]. The two kinds of influxers mentioned above are H^+^ symporters triggered by a proton gradient established by H^+^-ATPase on the plasma membrane. Some important genes related to Suc phloem unloading have been identified. For instance, *SbSUT1* and *SbSUT5* played an indispensable role in retrieving Suc from the apoplasms of Sorghum internodes to metaphloem sieve elements [29]. *OsSWEET11* and *OsSWEET15* are necessary for seed filling by exporting sugar from the maternal tissues to the apoplasm of embryos in *Oryza sativa* [8]. In maize and rice, CWINs performed a central function in assimilate unloading to developing seeds, which increased grain filling and crop yield [30,31]. Studies in tomato indicated that CWINs and HTs co-regulate the apoplasmic phloem unloading in ripening fruits [32,33,34]. 

Transcription factors are principal regulators in a variety of biological processes. Although how exactly TFs regulate phloem unloading-related genes is still unclear; some TFs involved in this process has been identified. A bZIP transcription factor ELONGATED HYPOCOTYL5 (HY5), was found to promote carbon assimilation and translocation, thus mediating assimilates unloading and shoot growth in response to light in Arabidopsis [35]. In cotton, GhMYB212 was involved in the regulation of sucrose transport into expanding fibers [36]. The overexpression of *MdAREB2* in apples activated the transcription of *MdSUT2* and finally affected fruit quality by the accumulation of soluble sugar [37]. OsDOF11 modulated sugar transport in rice by regulating the transcriptional expression of *SUT* and *SWEET* genes [38].

In this study, two different phloem unloading pathway stages were discerned by carboxyfluorescein (CF) transport under a confocal laser-scanning microscope. Given the technique difficulties to access the vascular tissues directly, we utilized transcriptome sequencing technology to profile a constructive landscape in *C. oleifera* pericarp and seeds from apoplasmic to symplasmic unloading pathway stages to explore the potential physiological and molecular mechanisms of the phloem unloading switch and identified a series of key genes and metabolic pathways likely participating in the shift of the phloem unloading route in oil tea fruit.

## 2. Results

### 2.1. Phloem Unloading Pathway Altered during the Late Period of C. oleifera Fruit Development

**Figure 1:**
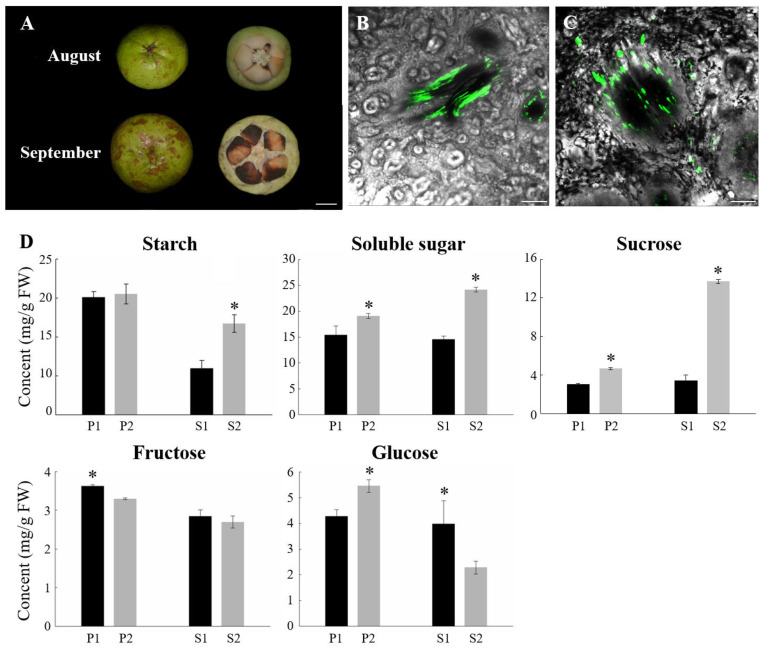
Physiological phenotype of oil tea fruit at two different developmental periods. (**A**) The fruit types used in the experiment. Bars = 1 cm; Confocal imaging of CF transport in phloem strands during oil tea fruit development. Transverse section of major vascular bundle in the fruit in August (**B**) and September (**C**). Bars = 250 μm; (**D**) Sugar concentrations in oil tea fruit at different stages. P1: pericarp in August; P2: pericarp in September; S1: seeds in August; S2: seeds in September. Bars represent the mean value of three replicates ± SD. Asterisk (*p* < 0.05) indicate significant differences between two sampling periods.

In the previous study, we reported the phloem-unloading pathway transition of sucrose in *C. oleifera* fruit. Sugar was transported via the symplast system in the early and late phases, compared to the apoplast system in the middle stage during *C. oleifera* fruit development [6]. To investigate the gene expression profile during the different phloem unloading stages, we first defined two stages of fruit development of the ‘Hua Shuo’ variety: middle stage (mid-August) and late stage (mid-September), which showed a distinct phloem unloading pathway and physiological characteristics (Figure 1A). The images of CF fluorescence in pericarp tissue showed that fluorescence in vascular bundles could be detected only in the phloem strands at mid-August. However, at mid-September, the dye diffused out of the phloem strands, being widely distributed in adjacent cells (Figure 1B,C). These findings confirmed that the phloem unloading pattern follows an apoplasmic route in the middle stage, but a symplasmic route in the late stage in fruit.

We further assayed the change in sugar concentration in pericarp and seeds of *C. oleifera* fruit at both stages. The concentrations of Suc, Glu, and soluble sugar in the fruit pericarp at the late stage were markedly higher than that of the middle stage; however, the Fru concentration reversed to Suc. The starch concentrations of pericarp were unchanged in those two stages. In seeds, the concentrations of starch, soluble sugar, and Suc were increased at the late stage compared to the middle stage, whereas the Glu concentrations were clearly decreased at the late stage. Additionally, the concentrations of Fru remained unchanged between the two stages in seeds (Figure 1D). These results suggest that the content of soluble sugar and starch whether in fruit or seeds were raised at the late stage, suggesting the strong sugar metabolism activity and transformation from sugar to starch or oil at the late fruit development stage, which was in accordance with the ripening features in *C. oleifera* fruit [6,39].

### 2.2. Transcriptional Profiling of C. oleifera Pericarp and Seeds at Different Stages of Phloem Unloading

**Figure 2:**
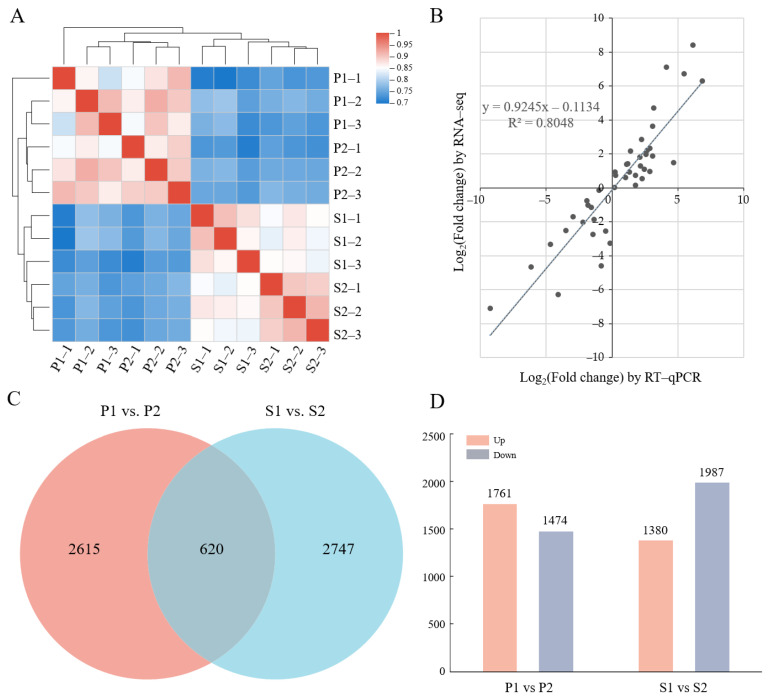
RNA-seq data and DEGs in apo- and symplasmic unloading stages of oil tea fruit. (**A**) Hierarchical clustering of 12 samples based on the correlation coefficient (r^2^) between each sample. The color panel represents the r^2^ values; (**B**) Correlation of expression changes observed by RNA-seq (*Y*-axis) and qPCR (*X*-axis); (**C**) Venn diagram of DEGs between fruit pericarp and seeds; (**D**) Statistics of up- and downregulated DEGs in pericarp and seeds for two different unloading periods. P1: pericarp in August; P2: pericarp in September; S1: seeds in August; S2: seeds in September.

To reveal the underlying mechanisms of phloem unloading and sugar transport in the *C. oleifera* fruit, we analyzed the transcriptional profiles of the *C. oleifera* fruit pericarp and seeds during the apoplasmic unloading-pathway stage and the symplasmic unloading-pathway stage. After filtered, a total of 96.65 Gb clean reads were obtained with more than 92.94% bases having a sequencing quality value of Q30. The alignment rates of the sequence mapping to the tea plant genome ranged from 74.53 to 84.46% (Table 1). A total of 66,261 expressed genes were identified, among which 52,014 (78.5%) genes were successfully annotated in at least one of the six protein databases (Table 2). Based on the correlation coefficient calculated with the Spearman algorithm, we performed a hierarchical clustering analysis for the samples. As shown in Figure 2A, the samples were clustered separately in two clades for the pericarp and seeds, and in the meanwhile, three replicates for each tissue were clustered together, which indicated the consistency between the gene expression profile and the corresponding sample or biological replicate. 

Given our emphasis on the comparison for apoplasmic and symplasmic unloading stages, we identified DEGs by subjecting the expression patterns to pairwise comparisons: P1 vs. P2 and S1 vs. S2. A total of 620 DEGs were induced both in the pericarp (P1 vs. P2) and seeds (S1 vs. S2), while 2615 and 2747 DEGs were specifically detected in the pericarp and seeds, respectively (Figure 2C). In the pericarp, the number of upregulated genes and downregulated genes at the late stage relative to the middle stage were 1761 and 1474, respectively. In seeds, compared with the middle stage, 1380 and 1987 genes were up- and downregulated at the late stage, respectively (Figure 2D). These findings suggested that the transcriptional responses to different Suc phloem unloading pathways were different in the pericarp and the seeds. With the development of fruit, more genes were transcriptionally upregulated in the pericarp, while the expression level of more genes decreased in seeds. To further elucidate the functions of the DEGs during the different phloem unloading routes in oil tea fruit, GO category enrichment analyses were performed using the DEGs shared in the seeds and the pericarp (Table S1). Genes associated with cellular components including the ‘cell wall’, the ‘apoplast’, the ‘plant-type cell wall’, and the ‘anchored component of plasma membrane’ were enriched. The enriched biological process included ‘DNA change’, the ‘auxin-activated signaling pathway’, the ‘carbohydrate metabolic process’, the ‘polysaccharide metabolic process’, and the ‘cell wall polysaccharide metabolic process’. In addition, some genes relevant to the molecular function of ‘hydrolase activity’ were also enriched (Table S1). 

To confirm the reliability of RNA-seq data, 19 genes were randomly selected for further expression analysis by qRT-PCR (Table S2). The high correlation coefficient (R^2^ = 0.8048) based on the expression changes observed by RNA-seq and qRT-PCR suggests that our RNA-Seq data are reliable (Figure 2B).

### 2.3. Screening of Differentially Expressed Structural Genes Involved in Phloem Unloading

**Figure 3:**
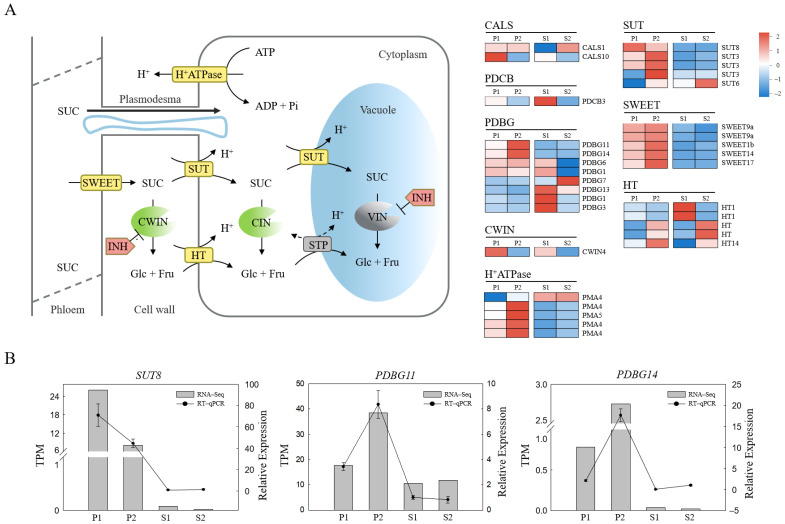
Expression of the differently expressed genes involved in phloem unloading of *C. oleifera*. (**A**) The current known model for principal transport steps and pathways for phloem unloading in *C. oleifera*. The heatmap of gene expression was generated from hierarchical cluster analysis of genes. Heatmap colors indicate the standardized expression level in each sample, and red and blue indicate from high and low expression, respectively, as shown in color bar on the right; (**B**) Gene expression patterns observed by RNA-seq (Y-axis on the left) and qPCR (Y-axis on the right). Error bars indicate SE (*n* = 3). P1: pericarp in August; P2: pericarp in September; S1: seeds in August; S2: seeds in September.

**Figure 4:**
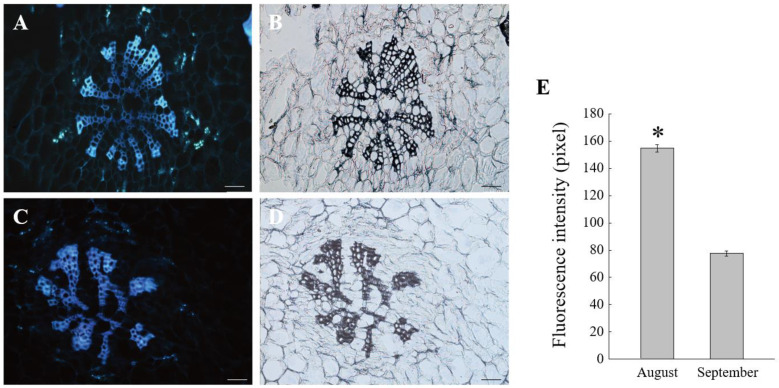
Labelling of cellulose in oil tea fruit pericarp. (**A**) Transverse section of major vascular bundle of oil fruit pericarp in Aug. and (**B**) is the corresponding micrograph in bright field; (**C**) Transverse section of major vascular bundle of oil fruit pericarp in Sep. and (**D**) is the corresponding micrograph in bright field; (**E**) Quantitative analysis of the florescent signal of cellulose in (**A**) and (**C**). Xy, xylem, Ph, phloem. Bar = 100 μm. Asterisk (*p* < 0.05) indicate significant differences between two sampling periods.

An increasing amount of evidence suggests that the metabolism of callose in the cell wall plays a central role in regulating PD permeability [23,40,41]. Genes involved in the regulation of callose metabolism in PD have been studied both in model plants and some fruit species [20,24]. In this study, we identified 11 differentially expressed genes involved in callose metabolism, consisting of two *CALSs*, one *PDCB,* and eight *PDBGs*. *CALS* and *PDCB* genes were upregulated in pericarp at the middle stage compared with the late stage, especially *CALS10* (*MSTRG.38744*). On the contrary, most *PDBG* genes were downregulated in the pericarp at the middle stage, especially *PDBG11* (*TEA009303*) and *PDBG14* (*TEA023721*) (Figure 3A). These results suggest that *CALS10*, *PDBG11*, and *PDBG14* are probably the candidate structural genes responsible for the change of unloading pattern by controlling the metabolism of callose deposited around the phloem in the pericarp. We further used the aniline blue staining assay for the visualization of oil tea fruit sections to observe the callose deposition in the phloem and the surrounding cells. Compared to the late stage, a stronger fluorescence was observed around the phloem sites in the middle stage, suggesting that more callose was deposited in the phloem region at the apoplasmic unloading period (Figure 4).

Considering the critical role of sugar transport proteins in the apoplasmic unloading mode, we identified DEGs encoding sugar transporters involved in phloem unloading, including five *SWEET* genes (*TEA010031*, *MSTRG.43542*, *TEA002811*, *TEA023624*, *MSTRG.16344*), five *SUT* genes (*TEA011262*, *TEA027283*, *TEA027286*, *MSTRG.62328*, *TEA032222*), and five *HT* genes (*TEA031246*, *TEA018089*, *TEA023599*, *MSTRG.46642*, *TEA002219*). In the pericarp, *SUT8* (*TEA011262*) was overexpressed at the apoplasmic stage whereas most of the other transport protein genes were highly expressed at the later stage. We also found a CWIN encoding gene *TEA004824* upregulated at the middle stage in the pericarp and the seeds. These results implied the potential roles of *SUT8* and *CWIN4* during the apoplasmic phloem unloading stage by conveying or catalysing the sucrose in the apoplast. 

Additionally, DEGs encoding H^+^-ATPase, which provides the proton gradient for the sugar transporter to uptake or release solute, were upregulated at the late stage in the pericarp (Figure 3A), in accordance with the most sugar transporter genes upregulating at this stage, which implied that these sugar transporters probably participated in the sugar retrieval and intracellular compartmentation at the symplasmic period. In seeds, two *HT* genes and *CWIN4* were highly expressed at the middle stage, while the other *HTs* and *SUT6* upregulated transcriptionally at the latter stage, showing their potential roles in sugar transport with seed development. The expression patterns of three candidate structural genes were confirmed with qRT-PCR, suggesting the reliability of our data (Figure 3B).

### 2.4. Differentially Expressed TFs and Their Co-Expression Network with Structural Genes

**Figure 5:**
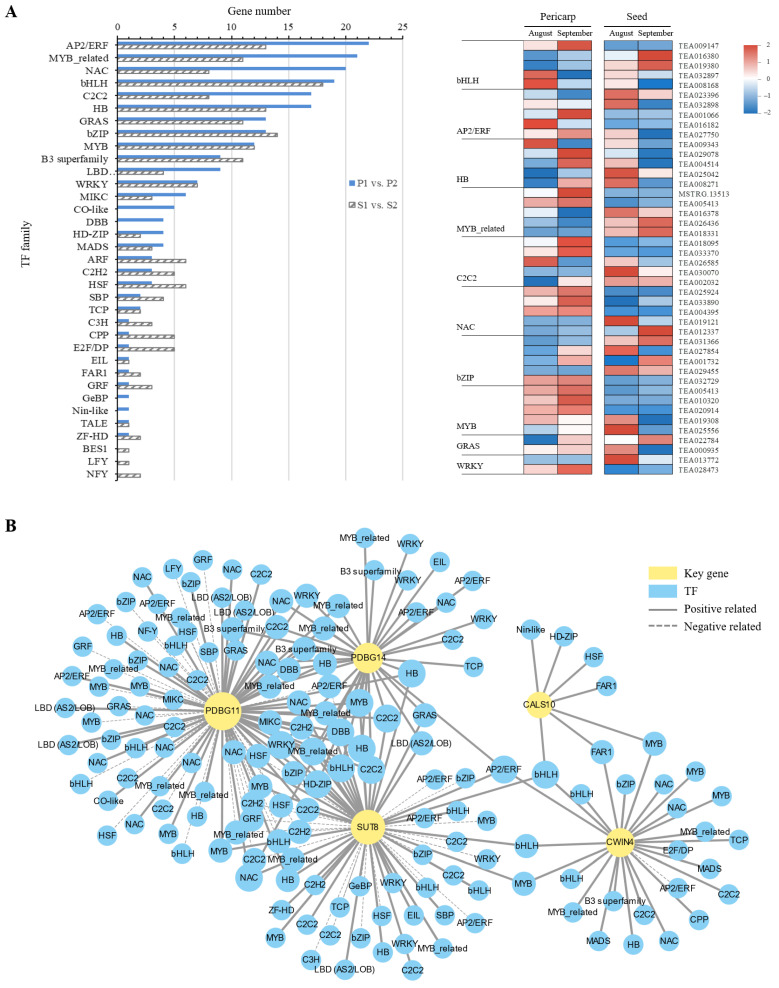
Differentially expressed transcription factors related to phloem unloading and their co-expression networks with candidate structural genes (**A**) Distribution of differentially expressed transcription factors (TFs) for the different unloading stages in pericarp (P1 vs. P2) or seeds (S1 vs. S2). Expression profiles of some TFs differentially expressed in fruit pericarp and seeds are shown in heatmap; (**B**) Co-expression networks between candidate structural genes in the phloem unloading and differentially expressed transcription factors (TFs). The width of the edge between each TF node and structural gene node represents the correlation coefficient.

A total of 2107 TFs from 44 families were identified from our RNA-seq data, of which 208 and 178 TFs distributed in 35 families were DEGs in the pericarp (P1 vs. P2) and the seeds (S1 vs. S2), respectively. In these TF families, the most DEGs belonged to the bHLH family, followed by the AP2/ERF family with 31 DEGs. A total of 41 TFs covering eighteen families were differentially expressed in both the pericarp and the seeds (Figure 5A). The Pearson product–moment algorithm was used to construct co-expression networks between differentially expressed TFs and the key structural genes identified. To improve the calculating precision, we calculated the correlation coefficient with a stringent cutoff (0.75) based on the gene expression data in all samples. It was found that SUT8, PDBG11, and PDBG14 were highly correlated since they shared more connected TFs in common (Figure 5B). These connected TFs included a MYB (*TEA010320*), two C2C2s (*TEA018095* and *TEA033370*), an NAC (*TEA004395*), a WRKY (*TEA028473*), and an AP2/ERF (*TEA016182*), etc. Our DEG analysis showed that *TEA010320*, *TEA018095,* and *TEA033370* were upregulated at the late stage in the pericarp, while *TEA016182* showed high expression levels at the early period in the pericarp (Figure 5A). In addition, the highly differentially expressed TF bHLH (*TEA008168*) was also highly correlated with *CWIN4*, *SUT8,* and *CALS10* in all samples. Collectively, these results help us construct a potential regulatory network of phloem unloading in oil tea fruit and these identified TFs probably participate in the phloem unloading pathway by regulating key structural genes.

### 2.5. Biological Processes Change with the Shift of the Phloem Unloading Route in Oil Tea

To identify biological pathways potentially relevant to the phloem unloading pathways in oil tea, KEGG enrichment analysis was performed. A total of 163 out of 620 DEGs identified collectively in the pericarp and seeds were enriched in 80 pathways, and the 18 most significantly enriched pathways are listed (Figure S1). These enriched metabolism pathways included ‘DNA replication’, ‘plant hormone signal transduction’, ‘starch and sucrose metabolism’, and pathways connected with fatty acid metabolism such as ‘biosynthesis of unsaturated fatty acids’, ‘alpha-Linolenic acid metabolism’, and ‘fatty acid degradation’, suggesting the active sugar metabolism, lipid biosynthesis with oil tea fruit development.

### 2.6. Plant Hormone Metabolism Responding to the Change of Phloem Unloading Pathways in C. oleifera Fruit

**Figure 6:**
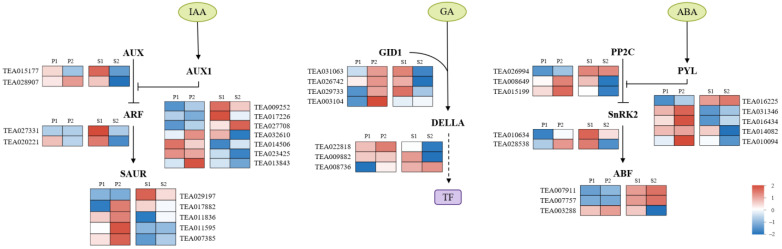
Heatmap of gene expression for DEGs enriched in the ‘plant hormone signal transduction’ pathway. Major enzymes in IAA, GA, and ABA signal transduction pathways are shown. Promotion or inhibition is indicated by arrows and blocks, respectively. Purple box and dotted arrow indicated the transcription factors and ubiquitination process, respectively. P1: pericarp in August; P2: pericarp in September; S1: seeds in August; S2: seeds in September.

Plant hormones play vital roles in carbon assimilate transport and distribution in plants [42,43]. In this study, 44 differentially expressed genes at the two unloading routes were identified to be involved in the ‘plant hormone signal transduction’ pathway including auxin (IAA), Gibberellin (GA), and abscisic acid (ABA) signaling pathways. We further compared the expression patterns of these genes in our RNA-seq data (Figure 6). In the IAA signaling pathway, DEGs showed the disparate expression patterns at the two phases. Specifically, *AUX1* and *SAUR* were highly expressed at the late stage compared to the middle stage, while the *AUX* and *ARF* showed almost no difference between the two phases in the pericarp. In the seeds, the expression of most IAA related genes was downregulated. DEGs in the gibberellin signaling pathway, including *GID1* and *DELLA*, were also identified. The expression levels of these genes were increased in the pericarp at the late stage relative to the middle stage but decreased in the seeds. Genes involved in the ABA signaling pathway such as *PYL*, *ABF*, *PP2C*, and *SnRK2*, had similar expression profiles in the pericarp that upregulated at the late stage relative to the middle period. 

### 2.7. Starch and Sucrose Metabolism Contributed to Sucrose Phloem Unloading in C. oleifera Fruit

**Figure 7:**
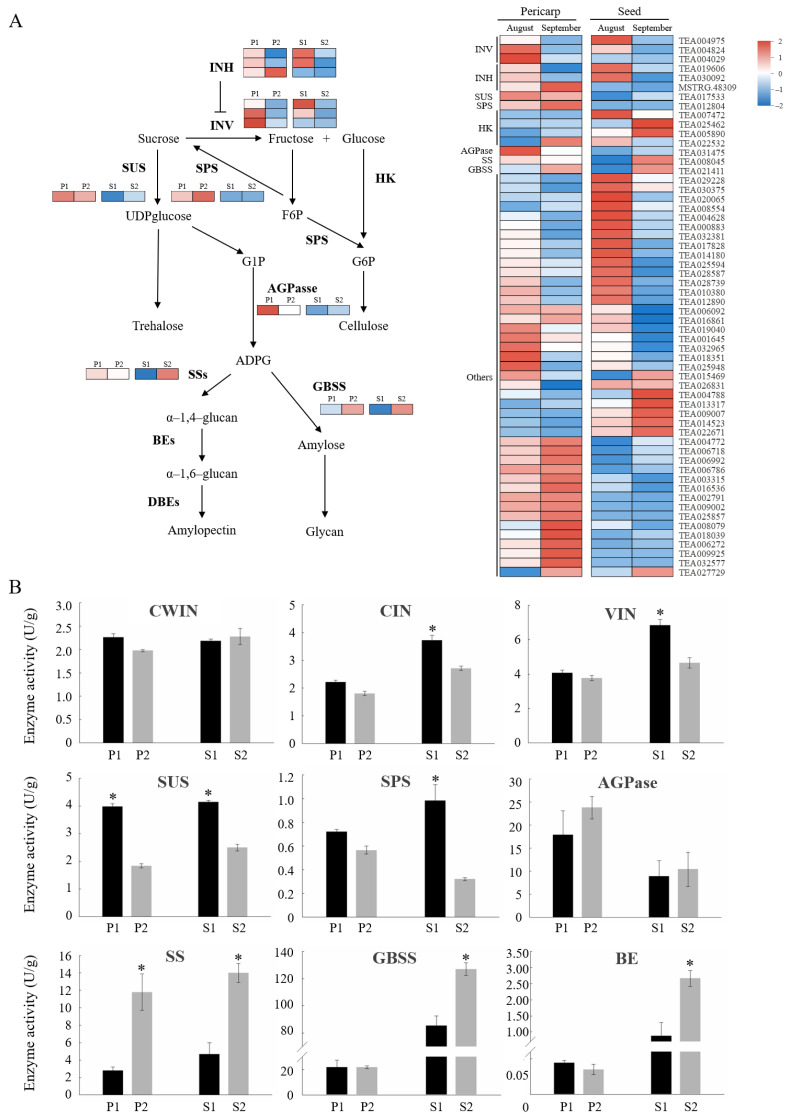
Expression pattern and enzyme activity of DEGs from the ‘Starch and sucrose metabolism’ pathway in oil tea. (**A**) Regulatory network of sucrose and starch metabolism underlying the DEGs in pericarp and seeds. Expression patterns of DEGs included in ‘Starch and sucrose metabolism’ pathway are shown in heatmap. (**B**) Enzyme activity of DEGs involved in sugar metabolism in oil tea fruit. Bars represent the mean value ± SE (*n* ≥ 5). The asterisk (*p* ≤ 0.05) indicates significant differences between two unloading stages. P1: pericarp in August; P2: pericarp in September; S1: seeds in August; S2: seeds in September.

The expression patterns of genes involved in the ‘starch and sucrose metabolism’ pathway at two unloading pathway stages were analyzed. The result showed that several important genes had different expression patterns at the two periods (Figure 7A). Genes connected to the sucrose cleavage, including sucrose synthase (SUS) and invertases (INV) such as *CWIN*, *CIN*, and *VIN*, were highly expressed at the middle stage, especially in the pericarp, while genes of the invertase inhibitor (INH) showed no difference in all the sample. The gene encoding sucrose phosphate synthase (SPS), responsible for sucrose synthesis, was sightly upregulated at the late phase in the pericarp but was not regulated in the seeds. Besides, a noticeable elevation in the expression levels of genes associated with starch synthesis was observed at the late period in seeds. We further determined the activity of enzymes mentioned above (Figure 7B). The enzymes implicated in sucrose metabolism exhibited higher activities at the middle stage than in the late period, particularly in seeds. The activity of SUS showed a rise at the middle stage compared to the late period both in the pericarp and seeds with the differences reaching a significant level. On the contrary, the enzyme activities involved in starch synthesis, including ADP-glucose pyrophosphorylase (AGPase), starch synthase (SS), granule-bound starch synthase (GBSS), and branching enzyme (BE) were higher at the late stage than the middle stage. SS activities at the late stage were more than four times as high as those at the middle stage in both the pericarp and seeds. These results suggest that the catabolic activity of sugar dominate in the pericarp during the apoplasmic unloading stage and a higher level of starch was accumulated at the late stage, particularly in the seeds, which is perhaps related to the sugar transformation at the late stage.

### 2.8. Lipid Metabolism-Related Pathways at the Two Developmental Stages of C. oleifera Fruit

**Figure 8:**
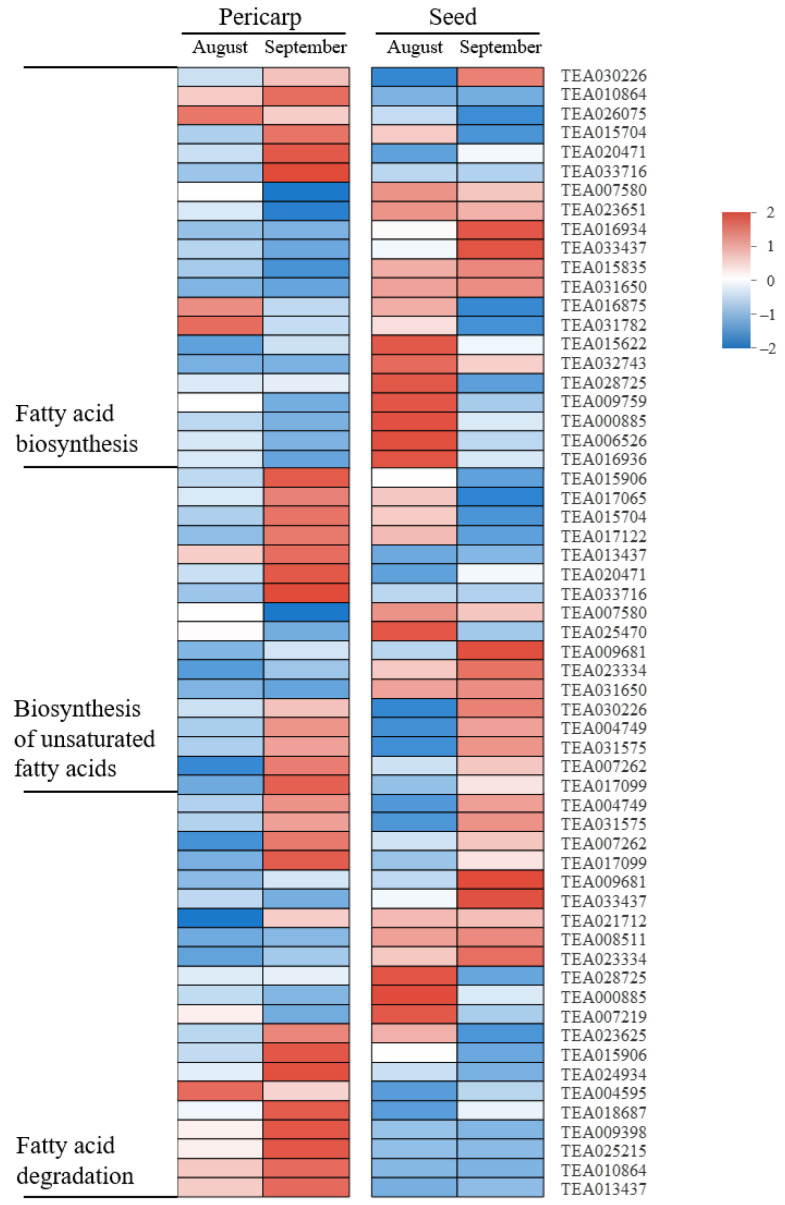
Gene expression for DEGs enriched in fatty acid metabolism related pathways. Heatmap colors indicate the expression level in each sample as shown on the right.

Oil is the main form of photoassimilate ultimately accumulated and utilized in mature *C. oleifera* fruits. We analyzed the expression patterns of DEGs involved in the lipid metabolism process including the ‘Biosynthesis of unsaturated fatty acids’, ‘Fatty acid biosynthesis’, and ‘Fatty acid degradation’ pathways both in pericarps and seeds (Figure 8). The results showed that the expressions of genes participating in these pathways mentioned above were almost upregulated in the late period relative to the middle stage in the pericarp. In seeds, there were a large number of lipid metabolism-relevant genes highly expressed in both periods. In addition, a DEG annotated to the sphingolipid metabolism pathway, *TEA024158*, was found to express more at the late stage than the middle stage in the pericarp but down regulate transcriptionally at the late development phase in seeds in our present study (Figure S2).

## 3. Discussion

Unlike fleshy fruit, the main harvesting and economic organ of oil crops such as *Camellia oleifera* is the seed rather than the pericarp. In fruit, the pericarp provides carbon assimilates as a temporary storage tissue and contributes a lot to crop yield by supplying maternal nutrients directly to the seed based on their natural physiological connection. Pericarp photoassimilate metabolism affected the oil content and the quality of seed [44]. In *Brassica napus*, carbon assimilation and sugar transport in the silique wall contributed to seed sugar concentration and lipid accumulation [45,46]. The sucrose phloem unloading pathway in the pericarp also changed with fruit development [15]. In addition, the unloading pathway transition of imported sugar was reported in many fleshy fruits [9,12,47], suggesting that it is a common phenomenon for the shift of the sugar phloem unloading route in fruit. However, little is known about the molecular mechanism of the sucrose phloem unloading transition.

In this study, the shift of the phloem unloading route in the variety ‘Hua Shuo’ was confirmed by CF labelling at the middle and late fruit development stages (Figure 1B,C). During the different phloem unloading stages, the content of carbon assimilates in fruit also changed. The contents of soluble sugar and starch increased at the late stage compared to the middle stage, especially in seeds (Figure 1D). Meanwhile, as reported by Zhang, et al. [48], the seed oil content also increased throughout development in ‘Hua Shuo’, implying the higher activity of storage nutrient accumulation and transformation processes at the late stage. Here, we performed deep transcriptome sequencing of the *C. oleifera* pericarp and seed samples at different unloading stages using RNA-seq. The transcriptome sequences were assembled into 66,261 genes, among which 78.5% were successfully annotated. A total of 2615 and 2747 differentially expressed genes were specifically detected in the pericarp and seeds, respectively. DEGs were mostly enriched in ‘DNA replication’, and ‘plant hormone signal transduction’ etc. Metabolism pathways and biological processes included the ‘carbohydrate metabolic process’ and the ‘cell wall polysaccharide metabolic process’. Our assembled and annotated C. oleifera genes provide a valuable resource for oil tea research and yield improvement.

By comparing the transcriptome profiles of *C. oleifera* fruits in this study, three structural genes in callose metabolism including *CALS10*, *PDBG11*, and *PDBG14* were identified to be differentially expressed at different unloading stages. Aniline blue staining showed that more callose was deposited in the phloem region at the apoplasmic unloading period than at the symplasmic stage (Figure 4). Since symplasmic phloem unloading usually involved functional PD connecting the surrounding cells, our results proved that the regulation of callose metabolism in the PD region around the phloem contribute to the change of the unloading pattern in oil tea fruit pericarp. Callose deposited at the PD neck region played a regulatory role in the neck switch [49]. The PD permeability was influenced by the synthesis and degradation of callose at the plasma membrane [23,40,41]. Callose synthase genes (*CALSs*) and callose degrading enzyme β-1,3-glucanase genes (*PDBGs*) are two antagonistic callose metabolic enzymes that regulate callose stability [50,51]. Therefore, in this study, we speculated that the upregulation of *CALS10* and reduced expressions of *PDBG11* and *PDBG14* in the pericarp at the middle stage (Figure 3) could reinforce the deposited callose to turn off the PD channel for isolation of the symplasmic connection between the phloem sieve elements and adjacent parenchyma cells in the pericarp. On the contrary, in the late phase of fruit development, the down regulated expression of *CALS10* and increased *PDBGs* expression contributed to the degradation of callose deposited around the phloem cells, consistent with the low level of callose determined by fluorescent labeling. Similar phenomena were also observed in other plant species. In cotton, the symplasmic connection between the fibre cells and seed coat was interrupted at the middle stage in cell elongation [52], which result from the callose being temporarily deposited at the PD neck regions [53]. 

A growing body of evidence has shown that apoplasmic phloem unloading in sink organs was regulated by SUTs [54]. In this study, differentially expressed *SUT* genes in oil tea fruit at two developmental stages were identified. Heatmap analysis showed that only *SUT8* was significantly upregulated at the middle developmental period, when the phloem unloading performed apoplastically, while the other *SUT* genes were significantly downregulated at the same phase in the pericarp (Figure 3A), suggesting the major role of SUT8 in apoplasmic phloem unloading. Up to date, multiple SUT family members have been widely identified in a variety of plants. In Arabidopsis, AtSUC2 was demonstrated to function in retrieving Suc leaked to the phloem apoplasm [55,56]. In addition to be transported directly into parenchyma cells by SUT, Suc unloaded in the apoplasmic space can also be degraded into fructose and glucose by CWIN and subsequently transported into sink cells by HTs in the resulting hexose. CWIN and sugar transporters both played important roles in carbon allocation and plant development [57]. Here, we also identified a gene *CWIN4* with a higher transcriptional level at the middle stage both in the pericarp and seeds (Figure 3A), suggesting its potential role in Suc apoplasmic unloading. The important role of CWIN in sucrose metabolism during oil tea fruit development was proven by enzymatic assay previously [39]. It has been recently reported that the interaction of INVs with INHs weakened the enzyme activity of INVs at a post-translational level [58]. Herein, we measured CWIN enzyme activities and found that there was no difference of CWIN activities between the two unloading stages, implying the possible existence of post-translational regulation of CWIN in oil tea fruit. This view was supported by the similar transcriptional expression patterns of INVs and INHs (Figure 7). Interestingly, we also found some differentially expressed sugar transport protein genes highly expressed at the late stage, including five *SWEETs* (*SWEET1b*, *SWEET14*, *SWEET17*, and two *SWEET9as*), three *HTs* (*HT14* and two *HTs*), and three *SUT3s* (Figure 3A), suggesting that they probably function in sugar transport at the symplastic unloading stage. As a matter of fact, in sweet Sorghum and sugarcane, SUTs participate in symplasmic unloading in stems by retrieving the Suc leaked in apoplasm back into SE–CC complexes of vascular bundles [29,59]. Since the late development of oil tea fruit was accompanied with a high concentration of soluble sugar, intensive starch accumulation, and oil transformation, we cannot exclude that besides the symplasmic unloading pathway, phloem unloading also operated apoplamically simultaneously at this stage to adapt to the vigorous sugar demand for carbon assimilate accumulation and transformation, and in the meanwhile, to hinder the potential back flow of sugar out from the fruit. The apoplasmic phloem unloading pathway may be a common mechanism to prevent backflow in the terminal strong storage sinks accumulating a high level of soluble sugars [60]. This opinion is further proven by the high enzyme activity of CWIN remaining at the late developmental stage. 

Sucrose cleavage was catalysed by invertase (INVs) or sucrose synthase (SUS), and INVs can be classified as cell wall INV (CWIN), vacuolar INV (VIN), and cytoplasmic INV (CIN) [61]. Suc transported into PCs can be hydrolysed by CIN or SUS, both of which had effects on the unloading and growth capacity of sink organs, thereby determining sink strength [62]. In apple [63] and kiwifruit [64], SUS was predominantly responsible for Suc degradation in apoplasmic unloading and its activity was highly correlated with the sink strength of storage organs. In *Xanthoceras sorbifolia* fruit, SUS acted as the key enzyme catalyzing Suc degradation in the post-unloading pathway whether in the pericarp or in the seed, while VIN and CIN play compensational roles in sucrose decomposition [15]. In fact, SUS was found to play a dominant role in sucrose decomposition in many biological processes including sugar unloading and sink strength [65]. In this study, the enzymatic assays showed that the activity of SUS in oil tea fruit pericarp was higher than that of CIN (Figure 7B) at the apoplasmic unloading period, implying the key role of SUS for hydrolysing cytoplasmic Suc in the apoplasmic unloading pathway in the pericarp, compared to CIN with relatively low activity at this stage. 

Plant hormones play important roles in regulating multiple physiological processes including photosynthate phloem unloading and accumulation in sink cells [43,66,67]. In this study, it was found that the hormone signaling was highly engaged in the regulation of symplasmic phloem unloading in oil tea fruits. In oil tea pericarp, the most genes involved in the plant hormone signal transduction pathway include auxin, gibberellin, and abscisic acid signaling pathways and these genes showed high transcriptional abundance in the late phase compared to the middle phase according to KEGG analysis, corresponding with the hormone content of fruits reported by a previous study [68]. For growth sinks, phytohormones could regulate membrane transport and Pdl conductance through calcium-connected signaling pathways [69,70], the latter of which functioned by altering callose deposition/hydrolysis [71,72] in the neck regions of PDs. This indicated that hormone-signaling pathways probably regulate the shift of the phloem unloading by restarting the symplasmic pathway via altering the PD permeability in oil tea fruit. In addition, in many kinds of fruits, ABA appear to be the key hormone regulating fruit ripening, which may also account for the elevated expression patterns of ABA-related genes. In particular, GA and IAA play important roles in promoting cell expansion growth [67,73]. Here, most GA and IAA signaling transcription-related genes were highly expressed at the middle phase compared with the late phase in seeds, which are consistent with the expansion and development of seeds in August. 

## 4. Materials and Methods

### 4.1. Plant Materials

*Camellia oleifera* var. ‘Hua Shuo’ were cultivated in the Wangcheng District, Changsha City, Hunan Province (latitude 28°05′ N, longitude 113°21′ E). The anthesis of this variety was from October to November each year and fruits ripened in late October of the following year. Four healthy trees were selected to collect the samples (pericarp and seed) at the middle and late developmental stages (mid-August and mid-September, respectively). Three biological replicates were performed for our collection, and each contained 8 fruits evenly collected from these trees. For convenience of delineation, we used P1 and S1 to represent the pericarp and seed samples in the middle stage, and P2 and S2 to represent the pericarp and seed samples in the late stage, respectively. Fresh samples were either used for paraffin section and fluorescent labeling or frozen with liquid nitrogen immediately and then stored at −80 °C for physiological measurements and RNA extraction.

### 4.2. Carboxy Fluorescein Diacetate Labeling

The experiment was carried out according to the method described by Zhang, et al. [60] with modification. A needle with thread was crossed through an Eppendorf tube with a wad of cotton put in it. Then put the needle through the phloem of pedicel and dropped approximately 150 μL of 1 mg/mL carboxy fluorescein diacetate (CFDA) solution into the tube. When loaded into fruit cells from pedicel by the thread, CFDA would been degraded into CF. After the dye was transported in the plant for 48–72 h, the fruit was removed immediately for free hand sections, including transverse and longitudinal sections. Microscopic examinations were conducted using a Leica SP8 confocal laser-scanning microscope under a blue light (488 nm).

### 4.3. Paraffin Sections and Fluorescent Labeling of Callose

The paraffin sections were made as described by Du, et al. [74]. First of all, fresh pericarps were fixed in FAA liquid (a 1:1:18 mixture (*v*/*v*/*v*) of formalin: acetic acid: 50% ethanol) immediately after being collected. Then, samples were dehydrated in a range of graded ethanol (70, 80, 95 and 100%). After being infiltrated with xylene/ethanol series (ethanol/xylene 1:1, and 100% xylene) and embedded in paraffin wax by conventional methods, samples were sectioned into slices of 12 μm using a rotary microtome (Leica RM 2265, Berlin, Germany). After sectioning, the slides were in xylene for 3 × 30 min and hydrated in ethanol series (100, 95, 85, 70, 50 and 30%) for 5 min. Three biological replicates per sample.

### 4.4. Paraffin Sections and Fluorescent Labeling of Callose

Callose of pericarp tissue sections from different developmental stages were stained with 0.05% aniline blue (Sigma, St. Louis, MO, USA) for 5 min. After incubation, sections were slowly washed with distilled water for 5 times and then dried in dark environment. The stained sections were observed under a biological microscope (Leica DM 2500, Berlin, Germany) with an excitation wavelength of 488 nm. The fluorescence intensity of callose in the phloem and surrounding cells was measured with Image-Pro Plus 6.0 software (Media Cybernetics Corporation, Rockville, MA, USA). Fluorescence intensity was measured at eight points around the phloem in each treatment, and final value was an average of the eight sets of data. Statistical data were processed with SPSS and statistical differences were determined by the one-way analysis of variance (ANOVA) method based on a t-test, with * *p* < 0.05 as significant.

### 4.5. Physiological Measurements

Freeze-dried fruit samples (0.1 g) were used for physiological measurements. The carbohydrate of fruit samples was extracted in 8 mL 80% ethanol (*v*/*v*) for 12 h, then the supernatants were used to determine the contents of total soluble sugar, glucose, fructose, and sucrose. The sediments were boiled for 3.5 h in 5 mL 2% HCl (*v*/*v*) and the supernatants were collected for measurement of starch content. The total soluble sugar, glucose, fructose, sucrose and starch contents were analyzed according to Jan’s report [75]. The enzyme activities of CWIN, CIN, VIN, SUS and SPS in fruit samples were determined as previously described by Li, Feng, and Cheng [63]. The activities of starch-related enzymes including AGPase, GBSS, SS, and BE in samples were measured as methods described by Jin, et al. [76]. Soluble proteins were measured with Coomassie blue [77], and enzyme activities were normalized on a protein basis.

### 4.6. RNA Extraction and Sequencing (RNA-Seq)

We used an OminiPlant RNA Kit (DNase I) (CWBIO, Beijing, China) to extract total RNA from the fruit samples of C. oleifera according to the manufacturer’s instructions. The concentration and purity of RNA were measured by Nanodrop 2000 (Thermo Fisher, Waltham, MA, USA). RNA integrity was evaluated via agarose gel electrophoresis and Agilent Bioanalyzer 2100 (Agilent, Folsom, CA, USA). About 1 μg of RNA was used to construct cDNA libraries. An Illumina HiSeq2000 platform was used for sequencing in a paired-end mode. The read length was 150 bp. The raw data were deposited to the National Center for Biotechnology Information (NCBI) as project number PRJNA817836.

### 4.7. RNA-Seq Data Processing and Analysis

After being filtered by removing adapters, reads containing poly (N) and low-quality sequences, a total of 96.95 Gb clean reads were obtained. Owing to the genome data of *Camellia oleifera* not being available yet, we mapped the clean sequencing reads to a tea plant (Camellia sinensis) reference genome (http://tpia.teaplant.org/download.html, accessed on 26 March 2019) using HISAT2 version 2.1.0 (https://daehwankimlab.github.io/hisat2/main/, accessed on 26 March 2019) with RefSeq annotations. Gene expression levels were calculated using RSEM version 1.2.31 (https://github.com/deweylab/RSEM/, accessed on 26 March 2019) by the TPM (Transcripts Per Million reads) method. Differentially expressed genes (DEGs) were identified based on |log2 (fold change)| ≥ 1 and false discovery rate (FDR) < 0.05. Genes were aligned to public protein annotation databases including NCBI-NR (NCBI Non-Redundant Protein Sequence Database), Swiss-Prot, Pfam, EggNOG (evolutionary genealogy of genes: Non-supervised Orthologous Groups), GO (Gene Ontology), and KEGG (Kyoto Encyclopedia of Genes and Genomes) (Table 2). GO enrichment analysis of DEGs was performed using Goatools software version 0.6.5. (https://github.com/tanghaibao/goatools, accessed on 26 March 2019). KEGG pathway enrichment analysis of DEGs was performed by R package.

### 4.8. Quantitative Real-Time PCR (qRT-PCR) Analysis

Approximately 1.0 μg of the extracted RNA was used to synthesize first-strand cDNAs with a 5X All-In-One RT MasterMix (with AccuRT Genomic DNA Removal Kit) (Applied Biological Materials Inc., Vancouver, BC, Canada). The qRT-PCR was performed on an ABI-StepOnePlu Real-time PCR System (ABI, London, UK), according to the manufacturer’s instructions, with PowerUp™ SYBR™ Green Master Mix (Thermo Fisher Scientific, Shanghai, China) [78]. GAPDH gene was used as a reference gene [79]. The relative transcript expression levels of each gene were calculated by the 2^−ΔΔCt^ method. All primers used for the selected genes were listed in Table S1. Three independent biological replicates were performed for data analysis.

## Figures and Tables

**Table 1:** The Sequence mapping after aligned to the genome of *Camellia sinensis*.

Sample	Total Reads	Total Mapped	Q30 (%)
P1_1	60,538,690	46,670,044 (77.09%)	93.14
P1_2	44,632,706	33,808,440 (75.75%)	93.09
P1_3	54,987,824	43,454,320 (79.03%)	92.94
P2_1	50,331,646	38,615,427 (76.72%)	93.19
P2_2	56,935,162	48,089,102 (84.46%)	93.43
P2_3	53,874,354	42,709,833 (79.28%)	93.21
S1_1	46,444,352	35,140,094 (75.66%)	93.55
S1_2	66,249,036	50,478,873 (76.2%)	93.58
S1_3	62,077,406	46,570,332 (75.02%)	93.6
S2_1	52,061,320	39,827,703 (76.5%)	93.67
S2_2	52,807,790	39,974,735 (75.7%)	93.73
S2_3	47,665,372	35,524,004 (74.53%)	93.1

**Table 2:** The number (percent) of genes successfully annotated to NCBI-NR, Swiss-Prot, Pfam, EggNOG, GO, and KEGG database.

Database Type	Gene Number (Percent)	Transcript Number (Percent)
GO	23,727 (0.3581)	43,987 (0.4111)
KEGG	30,413 (0.459)	48,065 (0.4492)
COG	49,385 (0.7453)	85,076 (0.795)
NR	51,682 (0.78)	88,293 (0.8251)
Swiss-Prot	39,734 (0.5997)	69,788 (0.6522)
Pfam	37,584 (0.5672)	66,079 (0.6175)
Total annotated	52,014 (0.785)	88,727 (0.8292)
Total	66,261 (1)	107,009 (1)

## Data Availability

RNA-seq data were submitted to https://www.ncbi.nlm.nih.gov/sra./PRJNA817836 (accessed on 21 March 2022) and the project number was PRJNA817836.

## Supplementary Materials

The following are available online at https://www.mdpi.com/article/10.3390/ijms23094590/s1.

## Abbreviations

ABA	Abscisic acid
ABF	ABA responsive element binding factor
ADP	Adenosine diphosphate
ADPG	Adenosine diphosphoglu
AGPase	ADP-glucose pyrophosphorylase
ARF	Auxin response factor
ATP	Adenosine triphosphate
AUX	Auxin-responsive protein IAA
AUX1	Auxin influx carrier (AUX1 LAX family)
BE	Branching enzyme
CIN	Cytoplasmic invertases
CWIN	Cell wall invertase
DBE	Debranching enzyme
DELLA	DELLA protein
F6P	Fructose−6−phosphate
Fru	Fructose
G1P	Alpha-D-Glucose 1-phosphate
G6P	Glucose−6−phosphate
GA	Gibberellic acid
GBSS	Granule-bound starch synthase
GID1	Gibberellin receptor GID1
Glc	Glucose
H^+^-ATPase	H^+^adenosine triphosphatase ATPase
HK	Hexokinase
HT	Hexose transporter
IAA	Auxin
INH	Invertase inhibitor
INV	Invertases
PP2C	Protein phosphatase 2C
PYL	Abscisic acid receptor PYR/PYL family
SAUR	SAUR family protein
SnRK2	Serine/Threonine-protein kinase SRK2
SPS	Sucrose phosphate synthase
SS	Starch synthase
STP	Sugar transport protein
SUS	Sucrose synthase
SUT	Sucrose transporters
UDPglucose	Uridine diphosphate glucose
VIN	Vacuolar invertases
