# Supplementary material for: Integrative Physiological and Transcriptomic Analysis Reveals the Transition Mechanism of Sugar Phloem Unloading Route in Camellia oleifera Fruit
Source: Int J Mol Sci. 2022 Apr 21;23(9):4590. doi: 10.3390/ijms23094590 (PMC9102078; doi:10.3390/ijms23094590)
Supplement: Supplementary file 1 [file ijms-23-04590-s001.zip › Supplementary Figure-revised.pdf]

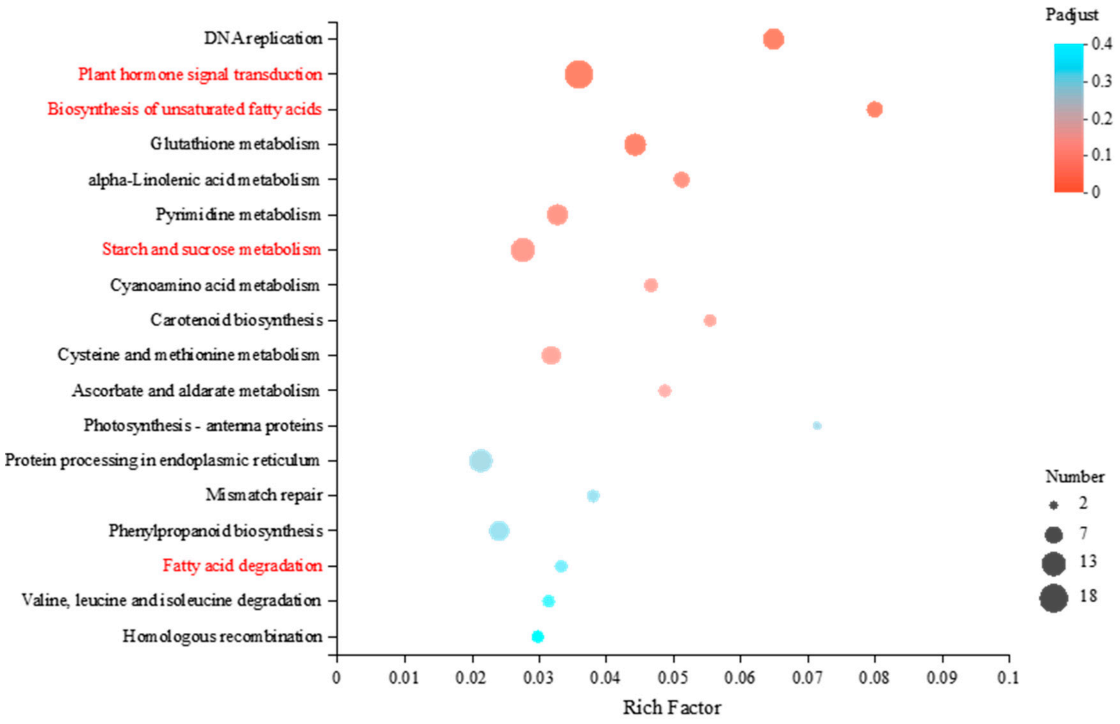

Figure S1 The 18 most significantly enriched KEGG pathways based on the coregulated DEGs in pericarp and seeds. The dot color and size indicate the P-value and gene number, respectively, as shown on the right.

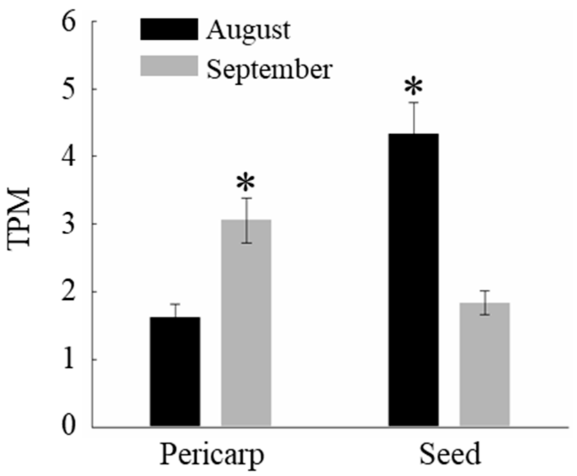

Figure S2 Transcriptional level of gene *TEA024158*.
